# Innovative methods for optimising clinical trial recruitment and retention within primary care

**DOI:** 10.1186/1745-6215-16-S2-P105

**Published:** 2015-11-16

**Authors:** Sarah Lawton, Simon Wathall

**Affiliations:** 1 Research Institute for Primary Care & Health Sciences, Keele University, Keele, Staffordshire, UK

## Background

Recruiting patients to research and collecting study data in a primary care setting, combined with maximising retention rates from a primary care population can be challenging and requires recruitment and retention methods which are innovative, efficient and transferrable.

## Objective

To apply low resource intensive and rapidly implemented recruitment and retention innovations for use in primary care settings that ensure patient recruitment and follow-up targets are achieved.

## Methods

A range of innovative recruitment and retention strategies are utilised by Keele CTU including; electronic aide-memoires linked to Read codes; physical aide-memoire prompts in consulting rooms; automated referral methods; postcard, repeat, email, SMS and minimum data collection reminder mailings; death and departure auditing.

## Results

Methods used; sustain routine care whilst simultaneously screening for research data and participants; provide flexible instruments compatible with all general practice infrastructures; increase clinical precision in identifying suitable participants; automates recording of study data collection; ensures minimal impact on consultation time; contribute towards the delivery of excellent retention rates.

## Conclusions

Recruitment aide-memoires, automated innovations and retention strategies can all be embedded easily into a primary care setting. These tools, without over burdening the busy primary care practitioner, result in simple and effective methods of prompting patient recruitment and retention. Excellent recruitment and retention rates are possible, despite encountering differences in primary care infrastructure. Such methods should be utilised more widely to facilitate primary care research, if this is where many conditions are diagnosed and managed.

